# Caregiver Well-Being and Burden: Variations by Race/Ethnicity and Care Recipient Nativity Status

**DOI:** 10.1093/geroni/igaa045

**Published:** 2020-09-15

**Authors:** Heehyul E Moon, William E Haley, Sunshine M Rote, Jeanelle S Sears

**Affiliations:** 1 Kent School of Social Work, University of Louisville, Kentucky; 2 School of Aging Studies, College of Behavioral and Community Science, University of South Florida, Tampa; 3 Department of Human Services, Bowling Green State University, Ohio

**Keywords:** Caregivers, National Health and Aging Trends Study (NHATS) and the National Study of Caregiving (NSOC), Nativity status, Race/ethnicity

## Abstract

**Background and Objectives:**

Despite growing diversity among the aging population and extensive previous research on racial/ethnic minority caregivers, little research has been conducted on the potentially unique experiences and outcomes of informal caregivers of foreign-born care recipients. Using nationally representative data and the Stress Process Model, the current study examined the differences in caregiver outcomes (care burden, psychological well-being, and self-rated health) by care recipient nativity status (U.S.-born vs. foreign-born) and the extent to which caregiver outcomes vary by care recipient nativity status and caregiver race/ethnicity (non-Hispanic white, non-Hispanic black, Hispanic, and Others).

**Research Design and Methods:**

The current study used Round 5 of the National Health and Aging Trends Study and the National Study of Caregiving (*N* = 1,436). We conducted ordinary least squares regression to analyze the differences in caregiver’s outcomes by care recipient nativity status and caregiver race/ethnicity and to investigate the impacts of the inclusion of caregiving factors (background factors, primary stressors, secondary stressors, and resources).

**Results:**

Regression analyses showed that only care burden significantly varied by care recipient nativity status after controlling for covariates. Caregivers of foreign-born care recipients reported a higher burden. However, when interactions of care recipient nativity status × caregiver race/ethnicity were introduced, non-Hispanic black and Hispanic caregivers of foreign-born care recipients were more likely to report better psychological well-being and self-rated health compared to their counterparts. Across caregiver groups, better caregiver–care recipient relationship quality and less caregiver chronic conditions were associated with less burden and better caregiver psychological well-being and self-rated health.

**Discussion and Implications:**

Care recipient nativity status and caregiver race/ethnicity may have complex effects on caregiving experiences. Given the observed significant interaction effects for caregiver psychological well-being and self-rated health, cultural factors may affect the extent to which these caregivers appraise their caregiving. Future research should delve into the appropriate ways to assess care stress as well as resilience among each caregiver group. Our results indicate the need for research, education, and practice that assess cultural and within-group differences among caregivers and inform needed changes to structural barriers.

**Translational Significance:** Caregivers of foreign-born older adults report more care burden than caregivers of U.S.-born older adults. These caregivers typically are the adult children of their care recipient and spend more time helping with daily activities and medical care than caregivers of U.S.-born care recipients. Non-Hispanic black and Hispanic caregivers of foreign-born care recipients were more likely to report better psychological well-being and self-rated health compared to their counterparts. Culturally appropriate assessments and interventions are needed to address the unique challenges and resilience of caring for foreign-born care recipients.

The population of foreign-born individuals in the United States is now a record of 14% and is predicted to increase to more than 17% by the year 2065 (Lopez et al., 2015). In 2010, 13% of those older than the age of 65 were foreign-born, a 70% increase from the previous 20 years (Greico et al., 2012). By 2050, the foreign-born older adult population is projected to be more than 16 million (Treas & Batalova, 2007). This demographic trend is predicted to continue due to both the aging of the existing foreign-born population in the United States and through ongoing admissions of older immigrants and refugees (Leach, 2009; Wilmoth, 2012). Given these projections, it is imperative to understand the unique circumstances faced by informal caregivers of foreign-born older adults, a group whose well-being is of great concern in the context of this demographic shift (Casado & Sacco, 2012; Lahaie et al., 2013; Pinquart & Sörensen, 2005).

To begin, many foreign-born older adults enjoy certain health advantages, including a reduced mortality rate (Borrell & Lancet, 2012; Cunningham et al., 2008; Dupre et al., 2012). Explanations for foreign-born health advantages include formal immigration screening processes, healthier lifestyles prior to migration, and self-selection processes whereby those with the highest levels of capability, social support, and resources are those who tend to migrate (Cunningham et al., 2008; Markides & Rote, 2019; Riosmena et al., 2017; Ro et al., 2016). Recently arrived immigrants may also underreport negative health conditions due to differences in perceptions or lack of diagnosis (Jasso et al., 2004). There is also evidence that immigrant-dense neighborhoods and large families may provide sociocultural resources that help immigrants age successfully (Markides & Rote, 2019). It is also possible that foreign-born adults return to their countries of origin when health problems arise, a hypothesis known as the “salmon bias” (Markides & Rote, 2019).

At the same time, health disadvantages are also documented among the foreign-born, including for infectious diseases, injuries, some types of cancers, diabetes, and associated complications (Cunningham et al., 2008; Kaushik et al., 2007). Where health disadvantages exist, explanations include the effects of migration and acculturation stress, occupational differences and hazard exposure, diminished social support, lack of access to health care and insurance, and lower rates of help-seeking behavior and health care utilization among the foreign-born (Choi, 2015; Cunningham et al., 2008; Min et al., 2005). Reasons for lower rates of health care utilization include systemic inequalities and cultural barriers including socioeconomic constraints (e.g., low education and physically demanding occupations), lack of resources (e.g., insurance), lack of information regarding available services, limited English proficiency, “factors related to stigma and marginalization” (Derose et al., 2007, p. 1262), availability of culturally appropriate services, and expectations regarding family care (Angel et al., 2014; Choi, 2015; Choi et al., 2015; Cunningham et al., 2008; DeNavas, 2011; Derose et al., 2007; Derr, 2016; Graham et al., 2009).

Due to a higher prevalence of functional disabilities in foreign-born older adults, for some, health advantages in mortality occur simultaneously with longer periods of disability and greater need for care in older age (Garcia et al., 2015; Markides & Rote, 2014–2015; Mehta et al., 2014). Foreign-born older adults needing care are more likely to live with and receive care informally through family and social networks compared to older adults born in the United States (Angel et al., 2014; Lahaie et al., 2013; Torres-Gil et al., 2005; Treas & Mazumdar, 2002). Previous research suggests that due to racial/ethnic disparities in morbidity and disability among older care recipients, racial and ethnic minority caregivers such as Hispanic and non-Hispanic black caregivers transition into the caregiving role earlier in the life course and remain in the caregiving role for a longer time compared to non-Hispanic white caregivers (Aranda & Knight, 1997; Coon et al., 2004; Moon et al., 2019). Moreover, Hispanic and non-Hispanic black caregivers experience more time-intensive and demanding caregiving careers (Rote & Moon, 2018).

Many first-generation immigrant (foreign-born) care recipients rely on support from second- and third-generation family caregivers, particularly women (Lahaie et al., 2013). A growing body of research indicates that challenges for later generations arise as a result of differences between caregiver and care recipient personal, familial, group, and cultural expectations and values such as filial responsibility and reciprocity, as well as levels of acculturation (Jackson et al., 2007; Mendez-Luck & Anthony, 2016; Miyawaki, 2015; Treas & Mazumdar, 2002). These factors not only inform caregiver’s meanings and motivations for care but also perceptions and responses to illness, aging, caregiver burden, and caregiving-specific coping strategies (Apesoa-Varano et al., 2015; Guo et al., 2019; Liu et al., 2008; Yeo et al., 2001).

Some studies (Gupta & Pillai, 2002) indicate, for example, that adherence to cultural beliefs and norms about family caregiving may lower burden. However, others reveal that strong familism does not always lead to better caregiving outcomes, including with regard to depressive symptoms (Robinson Shurgot & Knight, 2005) and social isolation (Mendez-Luck & Anthony, 2016). It is also possible that as subsequent generations become more acculturated, familism diminishes and caregivers receive less support from other family members, which affects both the intensity and burden of their role (Anthony et al., 2017). At the same time, the lack of culturally appropriate care options and medical mistrust may also limit help-seeking behaviors from racial and ethnic minority family caregivers (Sun et al., 2012).

Where noted cultural differences exist, these are also shared with other caregiver characteristics that intersect with racial and ethnic minority status. For example, Youn et al. (1999) found that Korean and Korean American caregivers reported higher levels of both familism and burden compared to white American caregivers, but not after controlling for demographic and health variables like gender and health. Lahaie et al., 2013 found that compared to second-generation and American-born caregivers, first-generation caregivers who were employed experienced less flexibility and accommodations in their work environments and were more likely to leave their positions as a result of their caregiver role (Lahaie et al., 2013). First-generation caregivers may additionally encounter unique challenges navigating health care systems and language barriers compared to second- and third-generation caregivers (Liu & McDaniel, 2015). In addition to this diversity, understanding of minority caregiver experiences is challenged by differences in expressions of burden and the availability of a few culturally and linguistically tailored measurement instruments (Anthony et al., 2017). These factors not only inform caregivers’ meanings and motivations for care but also perceptions and responses to illness, aging, caregiver burden, and caregiving-specific coping strategies (Apesoa-Varano et al., 2015; Guo et al., 2019; Liu et al., 2008; Yeo et al., 2001).

Given that minority older adults are living longer and requiring more care, more support will be needed for caregivers to maintain their own health and well-being (Arias et al., 2017; Redfoot et al., 2013). In particular, racial/ethnic minority care recipients who heavily depend on family care may have fewer people in their social networks, and fewer social resources, compared to non-Hispanic whites (Taylor et al., 2013). This may reinforce the higher level of care burden on caregivers of foreign-born older adults who have been marginalized at a systemic level (Choi, 2015; Choi et al., 2015; Derose et al., 2007).

Research using nationally representative data on the coping mechanisms and stressors involved in the life experience of caregivers of foreign-born care recipients in the United States is needed. This study offers one of the first examples of research to meet this gap. The questions and hypotheses posed here were informed by Pearlin et al.’s (1990) Stress Process Model (SPM). The SPM has been widely used to investigate how caregiver outcomes are influenced by caregiver and care recipient socioeconomic and personal characteristics, objective (e.g., activity of daily living dependency) and subjective (e.g., overload) primary stressors, and mediating/moderating conditions (e.g., coping and social support). Given the comprehensiveness and diversity of the SPM, it is well suited as a framework for the present study. However, it has not been used to investigate the stress process by nativity status, so given the comprehensiveness of the SPM, the current study expands the SPM to incorporate the role of care recipient’s nativity status. Based on available evidence, we addressed three hypotheses:

H1: Caregivers of foreign-born care recipients will report more care burden and poorer self-reported physical health and psychological well-being compared to caregivers of U.S.-born care recipients controlling for covariates.
H2: Caregiver race/ethnicity will have a significant interaction effect between care recipient’s nativity status and caregiver’s care burden, self-rated physical health, and psychological well-being.
H3: Background factors, stressors, and resources will mediate the conditional associations between care recipient’s nativity status and race/ethnicity for caregiver’s care burden, psychological well-being, and self-rated physical health.

## Method

### Sample

We used the National Health and Aging Trends Study (NHATS) and the National Study of Caregiving (NSOC). NHATS is a nationally representative study of Medicare beneficiaries older than the age of 65, which is sponsored by the National Institute on Aging (grant NIA U01AG032947) through a cooperative agreement with the Johns Hopkins Bloomberg School of Public Health. Data collection began in 2011 (*N* = 8,245) and the first replenishment sample was initially interviewed in 2015 (*N* = 8,334). Response rates were 71% in 2011 and 77% in 2015. The current study used Round 1 and Round 5 of NHATS to identify care recipient’s nativity status and race/ethnicity. Detailed technical notes regarding the study’s design and sampling are publicly available (Kasper & Freedman, 2015; Montaquila et al., 2012).

The NSOC is a sample of informal caregivers identified by the NHATS participants. NSOC collects information on how the caregiver helps the care recipient in the NHATS with everyday activities, along with information on the caregiver’s own health, family, and income by a 30-min telephone interview. We used NSOC 5, annual cross-sectional data with a response rate of 63.7% in 2015 (NSOC 5 *N* = 2,204; Kasper et al., 2016). We restricted our study population to the care recipients and caregivers who lived in the community, rather than in institutionalized settings due to possible differences in resources, stressors, or abilities between community-dwelling care recipients and those living in care facilities (Ewen et al., 2017; Wysocki et al., 2012). To identify the primary caregiver for a given care recipient, we counted the number of caregivers interviewed per older adult. If an older adult had one caregiver, we used his/her information. For care recipients with multiple caregivers, we identified the primary caregiver as the one who performed the most caregiving duties (based on hours per day) and used his/her information, eliminating other caregivers. Thus, our analyses included only those caregivers who provided the most care to a care recipient living in the community or in a nonnursing home/residential care setting (*n* = 768). The current study included a total sample of 1,436 caregivers.

### Measures

#### Independent variables

NHATS provided the nativity status of the participants. Foreign-born care recipients (= 1) are compared with those born in the United States (= 0). Caregiver’s race/ethnicity from NSOC is self-reported and proxy-reported and distinguishes among race/Hispanic ethnicity, including non-Hispanic white, non-Hispanic black, Hispanic, and Others (American Indian/Asian/Native Hawaiian/Pacific Islander/Other).

#### Control variables

We included a number of caregiver’s demographics (background factors), stressors, and resources that have been identified in previous studies using elements of the SPM (Pearlin et al., 1990) as important predictors of caregiver’s outcomes using NSOC Round 5.

##### Background factors.

These included gender (female = 0, male = 1), age, recorded caregiver’s relationship to care recipient (spouse/partner = 1, others [e.g., daughter, son, daughter in law, and son in law] = 0), and recorded caregiver education (more than high school = 1). The number of caregivers who self-reported health/cardiovascular conditions including heart disease, high blood pressure, arthritis, lung disease, cancer, stroke, and difficulties with seeing and hearing were included.

##### Stressors.

 Both primary and secondary stressors were included. For primary stressors, caregivers were asked about help with self-care activities (i.e., activities of daily living, ADLs) and household activities (i.e., instrumental activities of daily living, IADLs), help with medical care, and help with medical appointments and insurance. For help with self-care activities and household activities, caregivers were asked how often they assisted with (a) shopping, (b) chores around the home, (c) personal care, (d) getting around, (e) driving places, and (f) other transportation (e.g., shuttle and bus), with response categories from 1 (*Never*) to 5 (*Everyday*) (α = 0.71). To assess help with medical care, caregivers were also asked if they assisted with (a) keeping track of medicines, (b) taking shots or injections, (c) managing medical tasks, (d) exercise, (e) a special diet, (f) care for teeth, (g) care for feet, and (h) skincare wounds, with dichotomized response categories (1 = yes; α = 0.72). To assess help with medical appointments and insurance, caregivers asked if they helped with (a) making medical appointments, (b) speaking to/emailing medical providers, (c) changing/adding health insurance plans or prescription drug plans, and (d) handling other insurance matters, with dichotomized response categories (1 = yes; α = 0.69).

As secondary stressors, caregivers were asked about limited outside activities due to care work including (a) visiting friends/families, (b) religious service, (c) club meetings/group activities, (d) going out for enjoyment, (e) working for pay/at a business, (f) volunteer work, and (g) providing other care with dichotomized response categories (1 = yes; α = 0.82). Caregivers’ self-reported financial difficulty due to caregiving was coded as present or absent (1 = yes, 0 = no). Level of family disagreement over care (In general, how much has your family disagreed over the details of sample person’s care?) was assessed with one item with response categories from 1 (*very much*) to 3 (*not so much*).

##### Resources.

We included the relationship quality with the care recipient, informal support from friends and family, and formal support. To assess relationship quality, caregivers were asked how much they experienced different relationship characteristics (e.g., “How much does [the care recipient] appreciate what you do for him/her?”) with response categories from 1 (*not at all*) to 4 (*a lot*) (α = 0.66). Caregivers were also asked how much they (a) enjoyed being with the care recipient, (b) the care recipient argued with the caregiver, (c) the care recipient appreciates the caregiver’s care, and (d) the care recipient got on the caregiver’s nerves, with response categories from 1 (*not at all*) to 4 (*a lot*) (α = 0.68). To assess informal support, caregivers were asked if they had friends/families to (a) talk to, (b) help with daily activities, and (c) help the caregiver care for the care recipient, with dichotomized response categories (1 = yes; α = 0.66). To assess formal support, caregivers were also asked if they had (a) gone to a support group, (b) used any services to take some time away from helping, (c) received any training to help care, and (d) helped find paid helpers to do household chores or personal care with dichotomized response categories (1 = yes; α = 0.63).

#### Dependent variables

NSOC asked caregivers regarding care burden if they (a) were exhausted at night, (b) had more things to do than they could handle, (c) did not have time for themselves, and (d) got a routine going when the care recipient needed changes (reversely coded), with response categories from 1 (*not so much*) to 3 (*very much*). We summed these four items as an indicator of caregiver’s care burden (α = 0.75). Caregivers were asked about their self-rated physical health using a single NSOC question from 1 (*Poor*) to 5 (*Excellent*). In order to measure the caregiver’s psychological well-being, NSOC used validated Patient Health Questionnaire-2 (PHQ-2, e.g., feeling down/depression; Kroenke et al., 2003) and the Generalized Anxiety Disorder Scale-2 (GAD-2, e.g., feeling nervous/anxious; Kroenke et al., 2007). We summed four items from the PHQ-2 and GAD-2, with scores from 2 to 8 (α = 0.74).

### Analysis Strategy

First, we presented the main study variables by nativity status (Table 1). Second, we presented the ordinary least squares (OLS) regression model for caregivers’ self-reported care burden, psychological well-being, and self-rated health by care recipient’s nativity status and race/ethnicity (H1, Table 2). Third, we presented the interaction effects of care recipient nativity status by caregiver race/ethnicity for self-reported care burden, psychological well-being, and self-rated physical health (H2, Table 3, Model 1). Finally, we presented the results of the inclusion of background factors, primary and secondary stressors, and resources to Model 1 to assess any mediated moderation effects (H3, Table 3, Model 2). The estimated correlations between outcomes and covariates are available in Supplementary Table 1.

**Table 1.** Proportions/Means of Study Variables by Nativity Status (NSOC, *N* = 1,436)

Variables	Caregivers of foreign-born care recipients (*n* = 136)	Caregivers of U.S.-born care recipients (*n* = 1,290)
*CG background factors*		
Age (mean)**	55.05	59.67
Education (%)		
≥Some college	57	60
Relationship to CG (%)		
Spouse	30	34
No. of CG chronic conditions (mean)	1.46	1.85
CG race/ethnicity (%)		
Non-Hispanic white	19	60
Non-Hispanic black	20	30
Hispanic	39	4
Others	22	6
*CG primary stressors*		
Help with ADLs and IADLs (mean)***	17.58	15.51
Help with medical care (mean)***	3.09	1.83
Help with medical insurance and appointments (mean)*	2.07	1.63
*CG secondary stressors*		
Limited activities (mean)*	1.45	0.60
Financial difficulties due to caregiving (%)***	38	19
Family disagreement over care (mean)	1.65	1.54
*CG resources*		
Formal support (mean)	0.63	.45
Informal support (mean)	2.07	1.96
Relationship quality with CR (mean)***	12.39	13.47
*CG outcomes*		
Care burden (mean)***	7.49	6.25
Psychological well-being (mean)	13.33	13.72
Self-rated physical health (mean)	3.34	3.35

*Notes:* CG = caregiver; CR = care recipients; NSOC = National Study of Caregiving; weighted data. Standardized coefficients beta are presented.

*p < .05, **p < .01, ***p < .001.

**Table 2.** Main Effects of CR Nativity Status on Caregivers’ Care Burden, Psychological Well-Being, and Self-Rated Health (NSOC 5, *N* = 1,436)

	Care burden		Psychological well-being		Self-rated health	
Variables	Model 1	Model 2	Model 1	Model 2	Model 1	Model 2
Constants	6.25	9.34	13.72	11.02	3.48	2.61
CR nativity status (reference: U.S.-born)	0.18***	0.094*	−0.043	−0.23	−0.05	−0.11
CG race/ethnicity (reference: white)						
Non-Hispanic black		−0.24		0.01		−0.05
Hispanic		−0.07		0.10***		0.00
Others		−0.03		−0.02		0.01
*CG background factors*		0.07		0.03		0.11**
Age						
Education (reference: ≤ high school)						
≥Some college		−0.07*		0.18***		0.15***
Relationship to CR						
Spouse		−0.09**		0.05		0.07
No. of CG chronic conditions		0.00*		−0.18***		−0.45***
*CG primary stressors*						
Help with ADLs and IADLs		0.12**		−0.08*		−0.05
Help with medical care		−0.01		0.030		−0.02
Help with medical insurance and appointments		0.09		0.01		0.03
*CG secondary stressors*						
Limited activities		0.28***		−0.13***		−0.05
Financial difficulties due to caregiving (%)		−0.09*		−0.07*		0.04
Family disagreement over care		−0.01		−0.07*		−0.04
*CG resources*						
Relationship quality with CR		−0.20***		0.12***		0.10*
Informal support		0.08*		0.05		0.04
Formal support		0.09*		−0.04		0.01
*R* ^2^	0.03	0.31	0.00	0.14	0.00	0.24
*F*	20.39**	22.71***	0.83	7.29***	−0.17	23.15***

*Notes:* CG = caregiver; CR = care recipients; NSOC = National Study of Caregiving; weighted data. Standardized coefficients beta are presented except for constants.

*p < .05, **p < .01, ***p < .001.

**Table 3.** Moderating Effects of Care Recipient Nativity Status by Caregiver Race/Ethnicity on Caregivers’ Care Burden, Psychological Well-Being, and Self-Rated Health (NSOC 5, *N* = 1,436)

	Care burden		Psychological well-being		Self-rated health	
Variables	Model 1	Model 2	Model 1	Model 2	Model 1	Model 2
Constants	6.34	9.24	13.74	11.17	3.51	2.71
CR nativity status (reference: U.S.-born)	0.22*	0.11	−0.15*	−0.12*	−0.17*	−0.16*
CG race/ethnicity (reference: white)						
Non-Hispanic black (NHB)	−0.03	−0.02	−0.02	−0.01	−0.07*	−0.07*
Hispanic	−0.03	−0.02	0.06	0.08**	−0.02	−0.04
Others	−0.10*	−0.08***	−0.05	−0.04	−0.01	−0.01
NHB CG × Foreign-born CR	−0.08	−0.06	0.07*	0.07**	0.10*	0.08*
Hispanic CG × Foreign-born CR	−0.04	−0.08	0.04	0.08*	0.08	0.13*
Other CG × Foreign-born CR	−0.06	−0.08	0.09	0.07	0.10	0.09
*CG background factors*						
Age		−0.07		0.03		0.11*
Education (reference: ≤ high school)						
≥Some college		−0.07*		0.18***		0.14***
Relationship to CR						
Spouse		−0.09*		0.05		0.07
No. of CG chronic conditions		0.01*		−0.18***		−0.45***
*CG primary stressors*						
Help with ADLs and IADLs		0.12**		−0.09*		−0.07
Help with medical care		−0.02		−0.04		0.04
Help with medical insurance and appointments		0.08*		0.02		0.04
*CG secondary stressors*						
Limited activities		0.30***		−0.13***		−0.06
Financial difficulties due to caregiving (%)		−0.08*		−0.08*		0.05
Family disagreement over care		0.01		−0.07*		−0.04
*CG resources*						
Relationship quality with CR		−0.20***		0.12**		0.10*
Informal support		−0.18*		0.05		0.03
Formal support		0.09*		−0.05		0.00
*R* ^2^	0.05	0.33	0.01	0.15	0.012	0.24
*F*	8.4***	18.47***	1.72	6.39***	1.29	19.84***

*Notes:* CG = caregiver; CR = care recipient; NSOC = National Study of Caregiving; weighted data. Standardized coefficients beta are presented except for constants.

*p < .05, **p < .01, ***p < .001.

## Results

### Characteristics of the Study Sample Caregivers by Care Recipient Nativity Status

As given in Table 1, we present proportions and means of caregiver’s demographics and other study variables by care recipient’s nativity status. Our results revealed that caregivers of foreign-born care recipients reported higher primary stressors than their counterparts including help with ADLs and IADLs (*t* = 3.89, *p* < .01), help with medical care (*t* = 5.71, *p* < .001), and help with medical insurance and appointments (*t* = 2.05, *p* < .05). Caregivers of foreign-born care recipients reported a significantly higher level of limited activities due to care, and more than one third of caregivers of foreign-born care recipients indicated financial difficulties due to caregiving compared to 20% of caregivers of U.S.-born care recipients (χ ^2^ (1) = 30.33; *p* < .001). There was no significant difference in family disagreement over care between the two caregiver groups. We also observed significantly worse relationship quality with the caregiver among caregivers of foreign-born care recipients than caregivers of U.S.-born care recipients (*t* = −4.25, *p* < .001). In terms of demographics/background factors, more caregivers of foreign-born care recipients were younger (*t* = −2.07, *p* < .05) than their caregivers of U.S.-born care recipients.

### Multivariate Statistics

#### Care recipient nativity status

In order to address H1, testing the main effects of care recipient nativity status on caregiver outcomes controlling for covariates, we first present models from OLS of caregivers’ self-reported care burden, psychological well-being, and health by care recipient nativity status before and after adjusting for background factors, primary and secondary stressors, and resources. As displayed in Table 2, results in Model 1 showed that compared to the caregivers of U.S.-born care recipients, caregivers of foreign-born care recipients reported significantly more care burden. In the next step, we examined if the significant differences in Model 1 (Table 2) were added background stressors, primary stressors, secondary stressors, and resources. The coefficient for foreign-born care recipients was reduced but remained statistically significant for care burden when these factors were included after controlling for all the covariates (see Model 2). In these models, we also found that race/ethnicity is significantly related to psychological well-being with Hispanic caregivers reporting better psychological well-being than non-Hispanic white caregivers.

#### Interactions between care recipient nativity status and caregiver race/ethnicity

**Figure 1.**
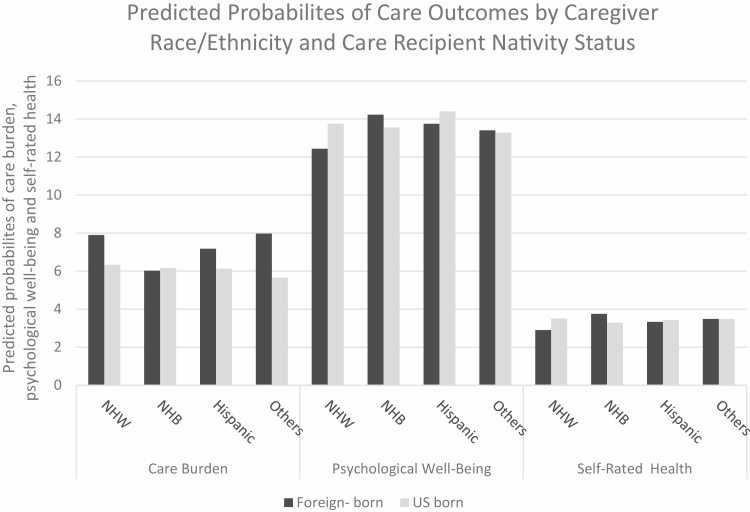
Predicted probabilities by caregiver race/ethnicity and care recipient nativity status adjusting for covariates. NHB = non-Hispanic black; NHW = non-Hispanic white; weighted data.

In order to address H2, we then identified the interaction effects of nativity status with race/ethnicity subgroups for caregivers’ care burden, psychological well-being, and self-rated health (Table 3). We found significant moderating effects of care recipient nativity status by race/ethnicity on caregiver psychological well-being and self-rated health (Model 1 of each outcome in Tables 3). For example, the coefficients of non-Hispanic black × care recipient nativity status for psychological well-being and self-rated health were statistically significant before adjusting for covariates. Figure 1 presents the predicted values of three outcome variables by care recipient nativity status × caregiver race/ethnicity subgroups before adjusting for covariates. As shown in Figure 1, for psychological well-being and self-rated health, non-Hispanic black caregivers of foreign-born older adults report higher well-being and better self-rated health compared to caregivers of U.S.-born older adults, which is an opposite pattern observed for the other groups.

Finally, in order to assess H3, we added background factors, primary and secondary stressors, and resources to Model 1 of Table 3 for each outcome. Model 2 of Table 3 for psychological well-being and self-rated health showed that the standardized coefficients for non-Hispanic black caregiver × care recipient nativity status were partially reduced but remained statistically significant. The results indicated that background factors, primary and secondary stressors, and resources do not fully explain better psychological well-being and self-rated health reported by non-Hispanic black caregivers of foreign-born care recipients than non-Hispanic white caregivers.

Also, as shown in Model 2 for psychological well-being and self-rated health in Table 3, we observed suppression effects of adding other covariates to Model 1 of each outcome because the coefficients of Hispanic caregivers × care recipient nativity status became statistically significant. In supplementary analyses (not shown), the coefficients of Hispanic caregivers × care recipient nativity status were not significantly associated with psychological well-being until background factors and primary stressors were added. As for self-rated health, the coefficients of Hispanic caregivers × care recipient nativity status became significant after adding background factors, and the significant association remained after adding primary stressors, secondary stressors, and resources.

**Figure 2.**
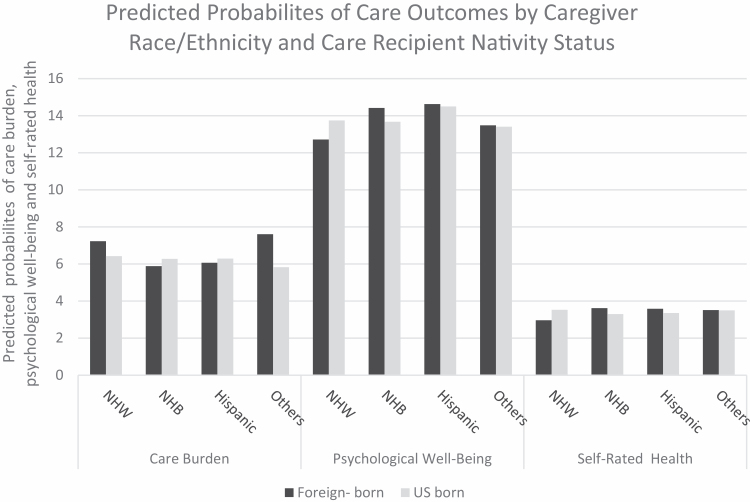
Predicted probabilities of care outcomes by caregiver race/ethnicity and care recipient nativity status after adjusting for covariates. NHB = non-Hispanic black; NHW = non-Hispanic white; weighted data.

Figure 2 shows the predicted probabilities of three outcome variables by care recipient nativity status × caregiver four race/ethnicity subgroups after adjusting for covariates to observe any changes after adding covariates. Non-Hispanic black and Hispanic caregivers of foreign-born care recipients reported less care burden than caregivers of U.S.-born older adults. Non-Hispanic white caregivers and other caregivers of foreign-born care recipients were more likely to experience care burden than their counterparts. For psychological well-being and self-rated health, non-Hispanic black and Hispanic caregivers of foreign-born older adults are more likely to report better psychological well-being and self-rated health than their counterpart caregivers. The relationship for non-Hispanic white caregivers, however, shows the opposite trend; caregivers of foreign-born care recipients reported worse psychological well-being and self-rated health than caregivers of U.S.-born care recipients.

## Discussion

To the best of our knowledge, this is one of the few studies in the United States to examine the role of care recipient nativity status on caregiver outcomes including primary caregiver’s care burden, psychological well-being, and self-rated health using nationally representative data. The major aim was to identify the extent to which caregiver’s care burden, psychological well-being, and self-rated health varied by care recipient’s nativity status and caregiver’s race/ethnicity.

### Caregiver Care Burden

We found that caregivers of foreign-born older adults report more care burden than caregivers of U.S.-born older adults. Previous studies on caregiving in immigrant families show that foreign-born care recipients tend to be more dependent on one focal child for help and less likely to rely on other relatives for late-life health support (Angel et al., 2014; Rote & Moon, 2018). Our results supported these findings in that caregivers of foreign-born older adults provided more help with ADLs and IADLs, medical care and medical insurance, and appointments than care recipients of U.S.-born older adults. More responsibility for these care tasks or providing care for older adults who need more assistance may account for a greater care burden; however, after we adjusted for primary stressors, secondary stressors, resources, and demographics, caregivers of foreign-born care recipients still displayed significantly higher levels of care burden than caregivers of U.S.-born older adults. Taken together, this means that caregiver burden is a persistent feature for caregivers to foreign-born older adults and may be more indicative of how caregivers of foreign-born older adults are coping with care demands than general health.

The current study only included the primary caregivers (who spent more time than other available caregivers) from the data and a large portion of care recipients are dependent on a daughter or son who may face multiple social roles (e.g., employment and child/other family care). Possibly, caregivers may experience or perceive more demands from their foreign-born care recipients compared to caregivers of U.S.-born older adults (Angel et al., 2014; Rote & Moon, 2018). However, when the interactions between care recipient nativity status and caregiver’s race/ethnicity and all covariates were considered, care recipient nativity status was no longer significant. In supplementary analyses (not shown), care recipient nativity status became insignificant when primary stressors were added to the analyses and remained insignificant until secondary stressors and resources were added. This suggests that caregivers of foreign-born care recipients may face high levels of primary stressors, and as previous studies show, fewer stressors and more resources may reduce the effects of care recipient nativity status or cultural differences on the care burden (Haley et al., 1996; Knight et al., 2000; Soskolne et al., 2007). The current study used the summed scores of burden, but future research should investigate any differences by care recipient nativity status for the different items of burden related to primary stressors (e.g., “had more things to do than they could handle”). Also, this study did not pay attention to shared caregiving responsibilities with other available caregivers. Given the higher care demands for primary caregivers (Wolff et al., 2018), having more available family caregivers contributing to care may reduce primary caregivers’ levels of care duties and perceived stressors (Lawrence et al., 2002; Roth et al., 2007). Future studies should also examine the extent to which the availability of family caregivers by care recipient nativity status is interrelated with care burden.

### Psychological Well-Being and Self-Rated Health

When caregiver race/ethnicity subgroups (non-Hispanic white, non-Hispanic black, Others, and Hispanic) were introduced to the analyses in the current study, we faced the complexity about the roles of caregiver stress by care recipient nativity status in psychological well-being and self-rated health. There were no significant roles of care recipient nativity status in caregiver psychological well-being and self-rated health before adding the interaction effects of care recipient nativity status and caregiver race/ethnicity. However, after adding interactions, the significant findings provide a further rationale for the investigation of subgroups differences in caregiving outcomes.

Our findings that non-Hispanic black and Hispanic caregivers of foreign-born care recipients were more likely to report better psychological well-being and self-rated health compared to their counterparts contradicts prior evidence that demonstrates lower levels of self-rated health and psychological well-being among racial/ethnic minority caregivers (Suwal, 2011). For non-Hispanic black caregivers, after adding the interactions with care recipient nativity status and caregiver race/ethnicity, care recipient nativity status became significant. Non-Hispanic black caregivers of foreign-born care recipients report better psychological well-being and self-rated health than non-Hispanic black caregivers of U.S.-born care recipients. Although the effect of care recipient nativity status and non-Hispanic black caregiver was attenuated, the significance remained when stressors, resources, and demographics were included. The findings suggest that, despite the significant roles of primary stressors, secondary stressors, and resources in caregiving outcomes (Haley et al., 1996; Knight et al., 2000; Soskolne et al., 2007), the role of interactions affects care outcomes differently among caregivers. As previous studies indicated, strong family support and a sense of obligation/commitment to caregiving among non-Hispanic black caregivers may posit caregiving as an embraced cultural value and norm with positive appraisals of the care of foreign-born care recipients with small or restricted social networks (Soskolne et al., 2007). This may occur even despite the high intensity of care provision among racial/ethnic minority caregivers (Friedemann et al., 2013; Rote & Moon, 2018). This may in turn lead to better psychosocial well-being and physical health.

For Hispanic caregivers, interactions with care recipient nativity status are nonsignificant for well-being or self-rated health unless background and stressors are included. When included, Hispanic caregivers of foreign-born care recipients report better psychological well-being and self-rated health. In supplementary analyses (not shown) each factor is added to the models separately. Results show that the interaction term for Hispanic caregivers by care recipient nativity status for self-rated health becomes significant with the inclusion of background factors. This indicates that when Hispanic caregivers of foreign-born care recipients are similar to caregivers of U.S.-born older adults on age, education, and number of health conditions, then they actually display an advantage in self-rated health. The interaction of psychological well-being reaches significance when secondary stressors are included such as limited activities and family disagreements.

Given that the interaction of Hispanic caregiver and care recipient nativity status becomes significant when controlling for background and secondary stressors, this suggests that if Hispanic caregivers of foreign-born care recipients were similar to their U.S.-born counterparts on these factors, they display a health advantage. As previous studies point out, there are several health advantages observed for foreign-born Hispanic adults in the United States. Foreign-born Hispanics tend to arrive in the United States in better health than their U.S.-born counterparts and migrate to immigrant-dense neighborhoods that can provide benefits and support to family caregivers (Rote et al., 2017). In the caregiving literature, familism is an important cultural value that prioritizes family well-being over individual’s preferences and can positively affect care outcomes (Aranda & Knight, 1997; Markides & Rote, 2019; Sayegh & Knight, 2011). Possibly, familism may shape the caregivers’ attitudes toward providing care as fulfilling filial duty and obligation and play a significant role in the caregiving process (Markides & Rote, 2019; Rote et al., 2015; Vega et al., 2011). However, there are many reasons Hispanic caregivers of foreign-born care recipients are at a disadvantage for these factors. First, Hispanic foreign-born care recipients tend to have smaller caregiving networks and be dependent on one caregiver for support in late life (Angel et al., 2014; Rote & Moon, 2018). Therefore, they may face secondary stressors, especially limitations of caregiving on activities due to less sharing of care work. Hispanic caregivers to foreign-born care recipients may also have different values about who is most responsible for care when compared to their foreign-born parents or family members. This is due, in part, to differences in cultural values in their country of origin compared to the country of destination and the unique stressors they face compared to caregivers of U.S.-born care recipients (Rote et al., 2019).

While these results are instructive, a noted limitation of this study is that the current study did not look at the long-term trajectory of changes in caregivers’ care burden, psychological well-being, and self-rated health, because as older adult’s conditions get worse, care burden and caregiver outcomes also change. The current study was also unable to investigate possible roles of cultural factors such as acculturation, length of stay in the United States, and fluency in English in caregiving due to the availabilities in data. The small sample size of foreign-born care recipients and the inclusion of other group, including American Indians and Asian Americans, may limit the generalizability of the study’s findings. The limited variability on race/ethnicity and relatively small sample size restrict the study’s generalizability to populations of color. Finally, the current study did not investigate the possible differences in caregiving by caregiver’s types of diseases, presence of disabilities, relationship to care recipient, and gender between caregivers and care recipients. Incorporating these variabilities in future research would further explain the observed differences in care outcomes among each group.

Despite limitations, our results contribute to current knowledge by highlighting differences and similarities in caregiving structures and is one of the few national studies demonstrating care recipient nativity status and caregiver racial/ethnic differences in caregiving. While all caregivers face challenges in meeting the demands of their care recipients, our results indicate the need for individualized approaches to practice with caregivers of foreign-born care recipients including sensitivity to cultural and within-group differences that include both risks and resilience factors (Dilworth-Anderson et al., 2020). There is a role, then, for practitioners, researchers, and educators to develop and evaluate culturally appropriate assessments and interventions for a diversity of caregiving groups. At the same time, our findings reinforce the understanding that structural and cultural forces shape the extent to which caregiving becomes stressful, as well as the quantity and quality of stressors and resources beyond the caregiving context, such as income, education, and a caregiver’s access to quality health and mental health care of their own. This implies the need for ongoing advocacy to address the systemic barriers that create and reinforce disadvantages for diverse families of foreign-born care recipients.

## Supplementary Material

igaa045_suppl_Supplementary_Table_S1
