# Supplementary material for: Caregiver Well-Being and Burden: Variations by Race/Ethnicity and Care Recipient Nativity Status
Source: Innov Aging. 2020 Sep 15;4(6):igaa045. doi: 10.1093/geroni/igaa045 (PMC7679974; doi:10.1093/geroni/igaa045)
Supplement: igaa045_suppl_Supplementary_Table_S1 [file igaa045_suppl_supplementary_table_s1.docx]

**Online Supplementary Material for *Innovation in Aging*:**

Moon, H., Haley, W. E., Rote, S. M., Sears, J. S. (2020). Caregiver well-being and burden: Variations by race/ethnicity and care recipient nativity status. *Innovation in Aging.*

Supplementary Table 1. Correlations of Caregiver Background Factors, Primary Stressors, Secondary Stressors, Resources, and Outcomes.

| Variables | Care Burden | Psychological Well-Being | Self-Rated Health |
| --- | --- | --- | --- |
| *Background Factors* |  |  |  |
| Age | -.07*** | .02 | .08** |
| Education (reference: ≤ high school) |  |  |  |
| ≥Some college | .02 | .08* | .02** |
| Relationship to CR (Spouse) | -.03 | -.01 | .07** |
| No. of CG Chronic Conditions | .02*** | -.12** | -.45** |
| *Primary Stressors* |  |  |  |
| Help with ADLs and IADLs | 0.28** | -.14** | -.08** |
| Help with Medical Care | .27* | -.06** | .00 |
| Help with Medical Insurance and Appointments | .28** | -.15** | -.10** |
| *Secondary Stressors* |  |  |  |
| Limited Activities | .48** | -.20** | -.14** |
| Financial Difficulties Due to Caregiving (%) | .17** | -.15** | .07 |
| Family Disagreement Over Care | -.21** | -.13** | -.06* |
| *Resources* |  |  |  |
| Relationship Quality with CR | -.32** | .22* | .16* |
| Informal Support | -.12** | .14** | .10** |
| Formal Support | .21* | -.05* | 0.22 |
| CR Nativity Status (reference: U.S. born) | .06** | .02 | .013* |
| CG Race/ethnicity (reference: White) |  |  |  |
| Non-Hispanic White | .01 | .00 | -.06* |
| Non-Hispanic Black | -.01 | .00 | -.06* |
| Hispanic | .22* | -.03 | -.01 |
| Others | -.03* | .04* | .03 |

Notes: NSOC = National Study of Caregiving; CG= caregiver; CR = care recipient; Weighted data. The correlation analysis provides the strengths and directions of the associations between outcomes variables and study variables.

*p < .05. **p < .01. ***p < .001.

u
